# Recent Hepatitis E Virus Infection in Wild Boars and Other Ungulates in Japan

**DOI:** 10.3390/v17040524

**Published:** 2025-04-04

**Authors:** Milagros Virhuez-Mendoza, Keita Ishijima, Kango Tatemoto, Yudai Kuroda, Yusuke Inoue, Ayano Nishino, Tsukasa Yamamoto, Akihiko Uda, Akitoyo Hotta, Hidenori Kabeya, Hiroshi Shimoda, Kazuo Suzuki, Tomoyoshi Komiya, Junji Seto, Yuki Iwashina, Daisuke Hirano, Mikio Sawada, Sayuri Yamaguchi, Fusayo Hosaka, Ken Maeda

**Affiliations:** 1 Department of Veterinary Science, National Institute of Infectious Diseases (NIID), Tokyo 162-8640, Japan; mvirhuez@niid.go.jp (M.V.-M.);; 2 Joint Graduate School of Veterinary Medicine, Yamaguchi University, Yamaguchi 753-8515, Japan; 3 Laboratory of Veterinary Food Hygiene, Department of Veterinary Medicine, College of Bioresource Sciences, Nihon University, Fujisawa 252-0880, Japan; 4 Hikiiwa Park Center, Wakayama 646-0051, Japan; 5 Faculty of Health and Medical Sciences, Hokuriku University, Kanazawa 920-1180, Japan; 6 Department of Microbiology, Yamagata Prefectural Institute of Public Health, Yamagata 990-0031, Japan; 7 Japan Wildlife Research Center, Tokyo 130-8606, Japan; 8 Livestock Hygiene Department, Aomori Prefecture Livestock Association, Aomori 030-0822, Japan; 9 Gifu Veterinary Medical Association, Gifu 500-8385, Japan; 10 Kagawa Prefecture Livestock Association, Takamatsu 760-0023, Japan; 11 Gunma Prefecture Livestock Association, Maebashi 379-2147, Japan

**Keywords:** hepatitis E virus, game meat, wild boar, sika deer, Japanese serow

## Abstract

Hepatitis E virus (HEV) is a zoonotic pathogen with multiple hosts, posing significant public health risks, especially in regions like Japan where game meat consumption is prevalent. This study investigated HEV infection and viral shedding in wild boars, sika deer, and Japanese serows across Japan. A total of 1896 serum samples were tested for anti-HEV antibodies, 1034 for HEV RNA, and 473 fecal samples for viral shedding. Anti-HEV antibodies were detected in wild boars from all seven prefectures studied, while HEV RNA was detected in wild boars from Fukuoka, Oita, and Miyazaki in southern Japan, as well as Yamaguchi prefecture. Genetic analysis revealed subtypes 3b, 4a, and 4g, with 3b being the most prevalent. Subtype 3b exhibited distinct geographical clustering, whereas 4g persisted exclusively in Yamaguchi for over 12 years. Infectious HEV particles were confirmed in wild boar feces, highlighting the risk of environmental contamination and zoonotic transmission. Sika deer showed no evidence of HEV infection, and only one Japanese serow tested positive for antibodies without detectable RNA. These findings underscore the importance of ongoing surveillance to assess the zoonotic risks from game meat consumption and prevention of HEV transmission to humans.

## 1. Introduction

Hepatitis E virus (HEV) is a single-stranded positive-sense RNA virus that causes an estimated 20 million infections worldwide [1]. HEV virions exist in two forms: quasi-enveloped particles in the bloodstream and non-enveloped particles in feces [2]. Belonging to the *Hepeviridae* family, HEV comprises four genera: *Paslahepevirus*, *Rocahepevirus*, *Chirohepevirus*, and *Avihepevirus* [3]. The *Paslahepevirus* genus includes two species, *P. alci* and *P. balayani*, with the latter comprising eight genotypes. Genotypes 1 and 2 are transmitted via the fecal–oral route, primarily through contaminated water, and exclusively infect humans, posing significant fetal, neonatal, and maternal mortality risks [4,5]. In contrast, genotypes 3 and 4 are zoonotic, primarily transmitted through foodborne routes, and are prevalent in industrialized countries. Although domestic pigs are the main reservoirs, these genotypes have been detected in various species and, more recently, in wastewater, shellfish, and seawater [6,7,8,9,10]. Genotypes 5 and 6 have been identified exclusively in wild boars in Japan, while genotypes 7 and 8 have been found in dromedary and Bactrian camels, respectively [11,12].

In Japan, genotypes 3 (HEV-3) and 4 (HEV-4) are the most prevalent, with transmission primarily occurring through zoonotic foodborne routes and, to a lesser extent, via blood or organ transfusions [13,14]. The first zoonotic case in Japan was linked to the consumption of sika deer [15]. Since then, HEV-3 and HEV-4 have been detected in various wild animals, including wild boars, mongooses, rabbits, and monkeys [16,17,18,19]. Additionally, HEV-3 has been detected in environmental samples, such as sewage and seawater, indicating its environmental circulation in Japan [20,21].

HEV exhibits broad host adaptability, infecting various ungulates, as evidenced by the detection of HEV RNA and anti-HEV antibodies in species such as moose, red deer, and roe deer [22,23,24,25]. While wild boars are widely recognized as the primary HEV reservoir in wildlife in Japan [12], the possibility of cross-species spillover infections and environmental transmission of HEV remains poorly understood. In Japan, where game meat consumption is widespread, wild ungulates represent a significant risk factor for HEV transmission to humans. Moreover, the recent increase in diagnosed HEV cases highlights the urgent need for continuous monitoring of HEV reservoirs and zoonotic transmission routes [13]. Therefore, this study aimed to assess HEV prevalence and viral shedding in wild ungulates across Japan to understand HEV circulation in wildlife and evaluate zoonotic transmission risks to humans.

## 2. Materials and Methods

### 2.1. Serum Sample Collection

In total, 1896 samples were collected from three species: wild boar (*Sus scrofa*), deer (*Cervus nippon*), and serow (*Capricornis crispus*), amounting to 952, 909, and 35 samples, respectively. Wild boar samples were collected from 2017 to December 2024 from seven prefectures, including Aomori (*n* = 13), Toyama (*n* = 57), Ishikawa (*n* = 173), Wakayama (*n* = 539), Yamaguchi (*n* = 51), Kagawa (*n* = 47), and Nagasaki (*n* = 72). Deer samples were collected between 2022 and 2024 from the prefectures of Aomori (*n* = 17), Gunma (*n* = 10), Gifu (*n* = 116), Wakayama (*n* = 328), Yamaguchi (*n* = 126), Kagawa (*n* = 30), and Nagasaki (*n* = 282). All samples from Nagasaki Prefecture were collected on Tsushima Island. A total of 35 serow samples were collected from Yamagata from 2017 to 2023. These wild animals were mainly captured as countermeasures under the official population control program. All samples were collected without overlap with those in our previous study [26]. Animal experiments were approved by the Japan National Institute of Infectious Diseases (NIID) Institutional Animal Care and Use Committee (Approval No. 122212).

### 2.2. Fecal Sample Collection

A total of 473 fecal samples were collected from wild ungulates across multiple prefectures in Japan, including 186 samples from wild boars and 287 from deer. Wild boar samples were collected from 2021 to January 2024 across 13 prefectures: Aomori (*n* = 10), Yamagata (*n* = 14), Chiba (*n* = 3), Toyama (*n* = 2), Shizuoka (*n* = 1), Nara (*n* = 4), Tottori (*n* = 1), Okayama (*n* = 3), Fukuoka (*n* = 6), Kumamoto (*n* = 7), Oita (*n* = 95), Miyazaki (*n* = 38), and Kagoshima (*n* = 2). Deer samples collected between 2021 and 2022 came from the prefectures of Hokkaido (*n* = 9), Aomori (*n* = 9), Iwate (*n* = 4), Yamagata (*n* = 1), Gunma (*n* = 7), Kanagawa (*n* = 1), Yamanashi (*n* = 3), Shizuoka (*n* = 39), Aichi (*n* = 7), Kyoto (*n* = 6), Osaka (*n* = 29), Hyogo (*n* = 7), Tottori (*n* = 26), Fukuoka (*n* = 1), Oita (*n* = 33), and Miyazaki (*n* = 54).

### 2.3. Sample Processing and Storage

Serum samples were transported to the laboratory in cooling boxes maintained at 4 °C and stored at −20°C. Fecal samples were diluted in phosphate-buffered saline (PBS) to a final concentration of 10% (*w*/*v*) and stored at −80 °C. They were subsequently clarified via centrifugation at 10,000× *g* for 1 min at 4 °C before RNA extraction.

### 2.4. Detection of Anti-HEV Antibodies in Wild Ungulate Sera

Anti-HEV antibodies in wild animal sera were detected using our previously reported highly sensitive enzyme-linked immunosorbent assay (ELISA) for various species [27,28]. Briefly, animal sera (100 µL per well) were added as the primary antibody in a 1:100 dilution, and detection was performed using peroxidase-conjugated protein AG (Thermo Fisher Scientific, Waltham, MA, USA). The absorbance was measured after adding ABTS 2-Component Microwell Peroxidase Substrate (SeraCare Life Sciences, Milford, MA, USA) and shaking the plates for 30 min at room temperature. The reaction was stopped with 100 µL of 1% sodium dodecyl sulfate, and absorbance was measured at 405 nm using a spectrophotometer (Bio-Rad, Hercules, CA, USA). As reported in our previous study on different mammalian species, the cut-off value was set at 0.437 for wild boar sera and 0.500 for other species [27,28].

### 2.5. HEV Genome Detection in Sera and Fecal Samples from Wild Animals

As previously described [29,30,31], RNA was extracted from fecal and serum samples using the MagMAX Pathogen RNA/DNA Kit (Thermo Fisher Scientific, Waltham, MA, USA) according to the manufacturer’s instructions. Due to the sample availability, 116 µL of serum and 116 µL of fecal samples were processed separately, resulting in a 50 µL elution each.

Nested reverse transcription polymerase chain reaction (RT-PCR) was performed to detect HEV RNA using the OneStep RT-PCR Kit (QIAGEN, Germantown, MD, USA) and KOD-Plus-NEO (Toyobo, Osaka, Japan). Primers targeting the conserved open reading frame 2 (ORF2) region of HEV genotypes 1, 3, and 4 were used for amplification, as previously described [27,32]. The resulting 378 bp amplicon was purified with the FastGene Gel/PCR Extraction Kit (Nippon Genetics, Tokyo, Japan), and sequencing was performed using BigDye Terminator v3.1 chemistry (FASMAC, Atsugi, Japan). Following primer removal, the final 338 bp sequences were deposited in the DNA Data Bank of Japan under accession numbers LC857165–LC857174.

### 2.6. Quantitative Real-Time RT-PCR (RT-qPCR)

The copy numbers of viral RNA were quantified using a one-step RT-qPCR assay with TaqMan Fast Virus 1-Step Master Mix (Applied Biosystems, Foster City, CA, USA) on a LightCycler 480 II (Roche, Vienna, Austria). A broadly reactive one-step RT-qPCR assay was performed using the forward primer JVHEVF (5′-GGTGGTTTCTGGGGTGAC-3′), reverse primer JVHEVR (5′-AGGGGTTGGTTGGATGAA-3′), and probe JVHEVP (5′-FAM-TGATTCTCAGCCCTTCGC-TAMRA-3′), following a previously described protocol [33,34]. The thermal cycling conditions were as follows: reverse transcription at 50 °C for 5 min, initial denaturation at 95 °C for 20 s, followed by 40 cycles of 95 °C for 3 s and 60 °C for 30 s. A 10-fold serial dilution of HEV-3 RNA (10^1^ to 10^7^ copies) was used to generate a standard curve for quantification [34,35].

### 2.7. Cell Culture and Virus Inoculation

The human hepatocarcinoma cell line PLC/PRF/5 (JCRB0406) was obtained from the Health Science Research Resources Bank, Japan. Cells were cultured in Dulbecco’s modified Eagle’s medium (DMEM) supplemented with 10% fetal bovine serum (FBS; Sigma-Aldrich, Saint Louis, MO, USA) and 1% penicillin-streptomycin (Fujifilm Wako Pure Chemical Corporation, Osaka, Japan), at 37 °C in a humidified 5% CO_2_ atmosphere.

For virus isolation, fecal samples were filtered through 0.45-μm filters (Corning, Corning, NY, USA). PLC/PRF/5 cells were seeded in 6-well plates (Sumitomo Bakelite, Tokyo, Japan) and inoculated with 100 μL of the filtered samples with 1% antibiotic-antimycotic solution (Gibco, Grand Island, NY, USA). The cells were incubated at 37 °C for 24 h. After 24 h, the medium was removed, and the cells were washed two times with DMEM. The culture medium was then replaced with fresh DMEM containing 2% heat-inactivated FBS and antibiotics. The medium was replaced every 3–4 days and supernatants were collected every 7 days for further detection of HEV RNA. Cells were observed daily for cytopathic effects.

### 2.8. Immunofluorescence Assay

HEK-293T cells were transfected with the plasmids pCAGGS-HEVcap (112–660) and pCAGGS using polyethyleneimine (PEI; Thermo Fisher Scientific, USA), as previously described [28]. Two days post-transfection, the cells were fixed with 10% formalin (Fujifilm, Osaka, Japan), permeabilized with 0.5% (*v*/*v*) Triton X-100 (Sigma-Aldrich, Saint Louis, MO, USA) for 30 min, and washed three times with PBS. Blocking was performed at 37 °C for 1 h using PBS containing 10% FBS, followed by three washes with PBS. Serow serum was used as the primary antibody at a dilution of 1:100 in PBS containing 1% FBS. For the positive controls, rabbit anti-HEV-1 VLPs hyperimmune serum (kindly provided by Dr. Tian-Cheng Li from the National Institute of Infectious Diseases) and monoclonal mouse anti-HEV ORF2 antibody (clone 1E6, Sigma-Aldrich, Saint Louis, MO, USA) were diluted at 1:1000 and 1:100, respectively. After 1 h of incubation at 37 °C, the cells were washed with PBS. For secondary staining, Protein A and G conjugated with Alexa Fluor 488 (Thermo Fisher Scientific, Waltham, MA, USA) was used for serow samples at a 1:100 dilution, while Alexa Fluor 488-labeled anti-rabbit IgG (Life Technologies, Chicago, IL, USA) and FITC-conjugated mouse IgG, IgM, and IgA antibodies (ICN Pharmaceuticals, Bryan, OH, USA) were used as positive controls. Fluorescence was visualized by indirect immunofluorescence microscopy, using pCAGGS-transfected cells as negative controls.

### 2.9. Phylogenetic Analysis

Phylogenetic analysis was performed using the MEGA7 software (version 7.0) based on partial ORF2 sequences (338 nucleotides). A phylogenetic tree was generated using the neighbor-joining method with 1000 bootstrap replicates, and branches with bootstrap values > 70% were grouped [36]. The analysis included reference sequences from the *Paslahepevirus* genus (genotypes 1 to 8), as well as the outer groups of *Rocahepevirus*, *Chirohepevirus*, *Avihepevirus,* and unclassified HEV sequences. For *Paslahepevirus* genotypes 3 and 4, the proposed subtypes were incorporated [36], including at least five strains per subtype when available, with the strains closest to our isolates available in NCBI up to January 2025 included in the analysis.

### 2.10. Statistical Analysis

All analyses, including the calculation of prevalence rates and corresponding confidence intervals, were performed in R version 4.4.3 (R Core Team, 2021). Chi-square analysis was used where appropriate, with statistical significance set at *p* < 0.05.

## 3. Results

### 3.1. Detection of Anti-HEV Antibodies and HEV RNA in Serum Samples of Wild Ungulates

A total of 952 wild boar serum samples were collected from seven prefectures, with 53 animals testing positive for anti-HEV antibodies, yielding an overall seroprevalence of 5.6% (95% confidence interval (CI) 4.20–7.22) (Table 1). The seroprevalence rates were 21.6% (11/51, 95% CI 11.29–35.32), 11.1% (8/72, 95% CI, 4.92–20.73), 10.6% (5/47, 95% CI 3.55–23.11), 7% (4/57, 95% CI 1.95–17.00), 7.7% (1/13, 95% CI 0.19–36.02), 3.5% (19/539, 95% CI 2.14–5.45), and 2.9% (5/173, 95% CI 0.95–6.62) for the Yamaguchi, Nagasaki, Kagawa, Toyama, Aomori, Wakayama, and Ishikawa prefectures, respectively (Table 2). In Yamagata, one of the 35 serow samples tested positive for anti-HEV antibodies, yielding a seroprevalence of 2.8% (95% CI 0.07–14.92). The positivity was confirmed using indirect immunofluorescence. All 909 deer serum samples from various prefectures were negative for anti-HEV antibodies (Table 1).

**Figure 1:**
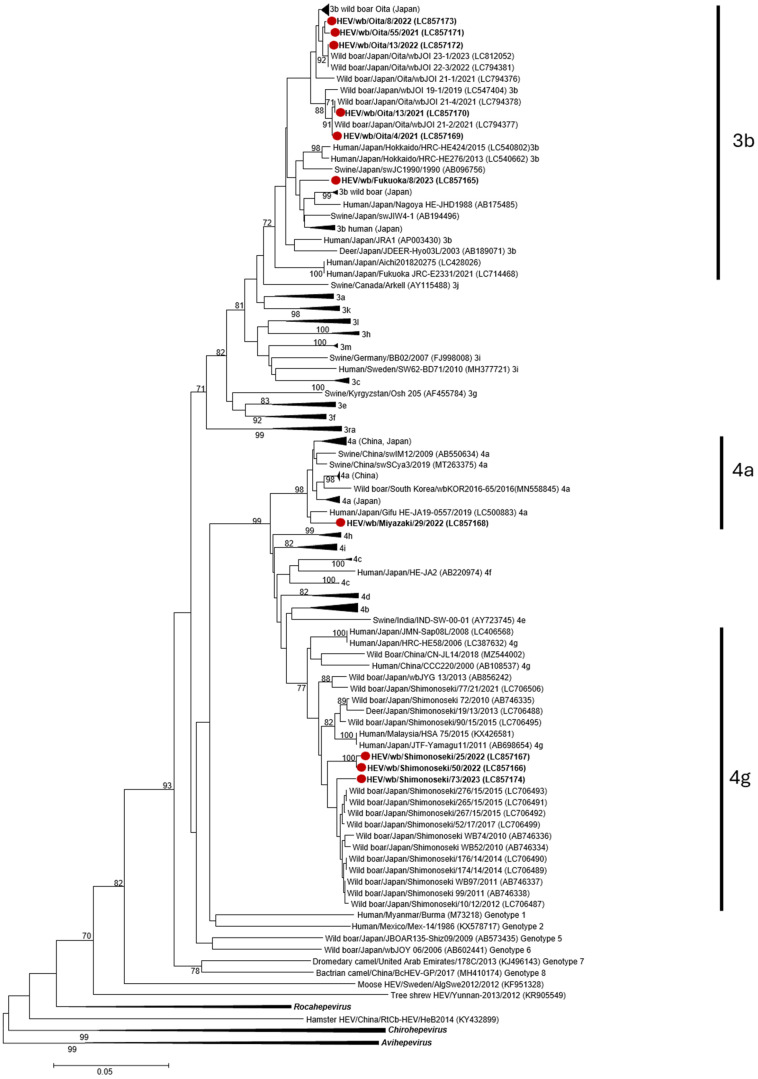
Phylogenetic analyses based on the partial ORF2 sequences (338 nucleotides) using the genotype 3 and 4 subtype reference strains proposed by Smith et al., 2020 [36] and the closest strains available in GenBank. The 10 wild boar strains obtained in the current study are highlighted with closed red circles (●). Subtypes 3b, 4a, and 4g are marked with vertical lines. Similar sequences are grouped by black triangles. For homogeneous groups, species and country of origin are indicated. The phylogenetic tree with 1000 bootstrap replicates was generated using the neighbor-joining method, and values less than 70% were removed. Sequences were labeled as “host/country/region/strain/year (GenBank accession number) subtype”. Scale bar indicates 0.05 nucleotide substitutions per site.

HEV genome detection was performed using nested RT-PCR, and HEV RNA was detected exclusively in wild boar samples collected from Yamaguchi Prefecture, with a positivity rate of 6.4% (3/47, 95% CI 1.34–17.54). All three detected strains belonged to genotype 4, subtype 4g (Figure 1). HEV RNA was not detected in deer serum samples. Serow samples were analyzed using nested RT-PCR and real-time RT-PCR to ensure the accuracy and sensitivity of our results, but no RNA was detected in either assay (Table 2).

### 3.2. HEV RNA Detection in Fecal Samples of Wild Boar and Deer

HEV genome detection in fecal samples was performed using nested RT-PCR, which showed positive results only in wild boar samples. The overall genome detection positivity rate was 3.8% (7/186, 95% CI 1.53–7.60) (Table 1). HEV RNA was detected in fecal samples from Fukuoka, Oita, and Miyazaki, with positivity rates of 16.7% (1/6, 95% CI 0.42–64.12), 5.3% (5/95, 95% CI 1.73–11.86), and 2.6% (1/38, 95% CI 0.07–13.81), respectively, whereas fecal samples from the remaining 10 prefectures were negative for genome screening (Table 3). Six of the detected strains belonged to genotype 3, subtype 3b, and one strain belonged to genotype 4, subtype 4a (Figure 1).

### 3.3. Infectivity of Wild Boar HEV Strains in PLC/PRF/5 Cells

Of the 10 strains, only the wb/Oita/8 strain (accession number LC857173) could infect PLC/PRF/5 cells. The cells were inoculated with 100 µL of the original stool suspension, which contained 2.39 × 10^8^ copies/mL of viral RNA. Viral RNA in the infected PLC/PRF/5 cells was first detected on day 7 post-infection (p.i.) at 1.73 × 10^4^ copies/mL. The viral load progressively increased to 5.93 × 10^6^ copies/mL by day 49 p.i. and reached 1.89 × 10^7^ copies/mL on day 84 p.i. (Supplementary Figure S1). To confirm the infectivity of the isolated strain, 0.5 mL of supernatant from day 84 p.i. was used to infect a 25T flask of PLC/PRF/5 cells, resulting in a viral RNA concentration of 3.25 × 10^6^ copies/mL by day 77 p.i. No cytopathic effects were observed in any of the passages.

## 4. Discussion

This study evaluated HEV infection and shedding in three wild ungulate species: wild boar, sika deer, and Japanese serow. We analyzed 1896 serum samples for anti-HEV antibodies, 1034 serum samples for HEV RNA, and 473 fecal samples from wild boars and deer for HEV RNA shedding. Anti-HEV antibodies were detected in wild boars across all seven prefectures, indicating that wild boars are likely the primary HEV reservoir in wildlife, as previously observed [26]. Seropositivity rates varied by prefecture, with Yamaguchi showing a rate of 21.6%, followed by Ishikawa (11.1%), Kagawa (10.6%), Aomori (7.7%), Toyama (7%), Wakayama (3.5%), and Nagasaki (2.9%).

Among the three ungulates, sika deer showed no evidence of HEV infection, with neither seroprevalence nor HEV RNA detected in serum or fecal samples, consistent with previous findings suggesting a low risk of HEV infection in this species [26,37]. In contrast, one Japanese serow tested positive for HEV antibodies using both ELISA and immunofluorescence assay; however, no HEV RNA was detected, making it difficult to conclusively classify the Japanese serow as a susceptible species for HEV infection. Although our HEV ELISA has demonstrated high specificity across various animal species [27,28], limited sample availability precluded further confirmation using western blot analysis. Other ungulate species, including roe deer, moose, red deer, and fallow deer [22,23,24,25,38,39,40,41,42], in Europe and Asia, have shown susceptibility to HEV infection. Additionally, antibodies against HEV but not HEV RNA have been reported in wild ruminants [43,44], suggesting that other mammals may be exposed to HEV. Although the absence of detectable HEV RNA in the Japanese serow limits definitive conclusions, the lack of previous research on HEV infection in this species highlights the importance of continued surveillance of wild ungulates to assess their susceptibility and role in HEV transmission.

The detection of HEV RNA in three wild boar serum samples and seven fecal samples from adjacent prefectures in southern Japan and Yamaguchi, including sub-genotypes 3b, 4a, and 4g, demonstrates the co-circulation of multiple HEV strains in wild boar populations. Sub-genotype 3b was the most frequently detected (6 out of 10 samples), which aligns with its established prevalence in Japanese swine, humans, and wild boars [13,45]. Subgenotype 4a, initially linked to China [36], has been identified in autochthonous Japanese cases since 2019 [13,46]. The 4a strain from Miyazaki wild boar clustered with other Asian isolates and more closely with a human-derived strain (LC500883) from a Japanese patient with a history of consuming wild boar and raw horse meat before HEV infection [46]. However, the strains shared only 95.86% identity, precluding confirmation of direct zoonotic transmission. Interestingly, subgenotype 4g, which is currently prevalent in humans in Japan, has only been detected in wild boars from the Yamaguchi prefecture [26,47]. The three 4g strains from wild boars clustered with the historical strains, indicating their persistence in the Yamaguchi prefecture for over 12 years. The absence of 4g strains in wild boars elsewhere in Japan suggests a geographically restricted circulation, underscoring the ability of HEV strains to establish long-term wildlife reservoirs.

Phylogenetic analysis revealed distinct clustering patterns within subtypes, reflecting geographical and host-specific variations, as observed in the distinct clusters formed by the 3b subtype strains from the adjacent Oita and Fukuoka prefectures in the Kyushu district. The 3b strains from Oita, detected in fecal samples, clustered with previously detected wild boar strains circulating in the same area [48], suggesting a unique strain endemic to Oita wild boars. These Oita strains did not cluster with a previously identified 3b strain isolated from deer in the same prefecture, indicating that the two strains may be spread among wild animals in Oita. In contrast, the 4g strains identified in this study grouped closely with the endemic Yamaguchi prefecture 4g strains from humans, deer, and wild boars, suggesting that cross-species transmission must occur in this region. These findings underscore the complex dynamics of HEV circulation in wildlife.

The successful isolation of the genotype 3b strain from a wild boar fecal sample, achieving a viral concentration of 1.89 × 10^7^ copies/mL by 84 days p.i along with the successful passage of the progeny virus, confirms the presence of infectious HEV particles in wild boar feces. This finding suggests that wild boars may contribute to environmental contamination, potentially exposing other wildlife and natural habitats, and could pose a risk to hunters handling game meat, as previously documented in studies on meat handlers [44,49,50].

Detection of anti-HEV antibodies and HEV RNA in serum and fecal samples from wild boar did not reveal a clear association between HEV infection and animal sex, in contrast to the gender-influenced HEV infection observed in human populations [14,45]. Additionally, wild boars weighing less than 30 kg showed lower rates of seroconversion than adults (>50 kg), with one animal exhibiting viremia and two showing fecal shedding (Supplementary Table S1). These findings highlight the importance of assessing the risk of HEV transmission from wild boars, particularly piglets.

This study showed the circulation of multiple HEV subtypes among wild boars in adjacent prefectures of southern Japan, with viral shedding detected in feces and antibodies identified in wild boars and a Japanese serow. Endemic strains were observed in wild boar populations, indicating that wild boars may be an important reservoir of HEV in wildlife in Japan. The emergence of new HEV subtypes and their host adaptation, along with the stronger association of genotype 4 with fulminant hepatitis than genotype 3 [51], highlights the need for continuous surveillance of wildlife. Genotyping and monitoring HEV in wild animals are essential for tracking disease dynamics and assessing zoonotic risks from game meat, ultimately guiding strategies to prevent HEV transmission to human populations.

## 5. Conclusions

Our study demonstrates the circulation of multiple HEV subtypes among wild boars in southern Japan. Infectious HEV particles were detected in wild boar feces, and antibodies were identified in both wild boars and a Japanese serow, although HEV RNA was not detected in the serow. These findings emphasize the importance of continuous surveillance and a one-health approach for managing the potential risk of zoonotic diseases.

## Figures and Tables

**Table 1:** HEV prevalence in wild ungulates in Japan.

Samples	Species	Collection Year	% of Anti-HEV Antibody-Positive Animals (No. of Positive Animals/No. of Examined Animals)	% of HEV RNA-Positive Animals (No. of Positive Animals/No. of Examined Animals)
Serum	Wild boar	2017–2024	5.6 (53/952)	0.4 (3/703)
	Deer	2022–2024	0 (0/909)	0 (0/296)
	Serow	2017–2023	2.8 (1/35)	0 (0/35)
Feces	Wild boar	2021–2024	-	3.8 (7/186)
	Deer	2021–2022	-	0 (0/287)

**Table 2:** Detection of anti-HEV antibodies and HEV RNA in serum samples.

Species	Prefecture	Collection Year	% of Anti-HEV Antibody-Positive Animals (No. of Positive Animals/No. of Examined Animals)	% of HEV RNA-Positive Animals (No. of Positive Animals/No. of Examined Animals)
Wild boar	Aomori	2022–2023	7.7 (1/13)	0 (0/13)
	Toyama	2022–2024	7 (4/57)	0 (0/57)
	Ishikawa	2017–2023	2.9 (5/173)	ND *
	Wakayama	2022–2024	3.5 (19/539)	0 (0/539)
	Yamaguchi	2022–2024	21.6 (11/51)	6.4 (3/47)
	Kagawa	2022–2024	10.6 (5/47)	0 (0/47)
	Nagasaki	2022–2023	11.1 (8/72)	ND
Deer	Aomori	2022–2023	0 (0/17)	0 (0/17)
	Gunma	2022	0 (0/10)	0 (0/10)
	Gifu	2022–2023	0 (0/116)	0 (0/116)
	Wakayama	2022–2023	0 (0/328)	ND
	Yamaguchi	2022–2024	0 (0/126)	0 (0/123)
	Kagawa	2022–2024	0 (0/30)	0 (0/30)
	Nagasaki	2022–2023	0 (0/282)	ND
Serow	Yamagata	2017–2023	2.8(1/35)	0 (0/35)

* ND: No data.

**Table 3:** Detection of HEV RNA in fecal samples.

Species	Prefecture	Collection Year	% of HEV RNA-Positive Animals (No. of Positive Animals/No. of Examined Animals)
Wild boar	Aomori	2023	0 (0/10)
	Yamagata	2021–2023	0 (0/14)
	Chiba	2023	0 (0/3)
	Toyama	2023	0 (0/2)
	Shizuoka	2021	0 (0/1)
	Nara	2021–2022	0 (0/4)
	Tottori	2021	0 (0/1)
	Okayama	2022	0 (0/3)
	Fukuoka	2022–2023	16.7 (1/6)
	Kumamoto	2021–2023	0 (0/7)
	Oita	2021–2023	5.3 (5/95)
	Miyazaki	2021–2023	2.6 (1/38)
	Kagoshima	2023–2024	0 (0/2)
Deer	Hokkaido	2021–2022	0 (0/9)
	Aomori	2021–2022	0 (0/9)
	Iwate	2022	0 (0/4)
	Yamagata	2022	0 (0/1)
	Gunma	2021	0 (0/7)
	Kanagawa	2022	0 (0/1)
	Yamanashi	2022	0 (0/3)
	Shizuoka	2021–2022	0 (0/39)
	Aichi	2022	0 (0/7)
	Kyoto	2022	0 (0/6)
	Osaka	2021–2022	0 (0/29)
	Hyogo	2022	0 (0/7)
	Tottori	2021	0 (0/26)
	Fukuoka	2022	0 (0/1)
	Oita	2021–2022	0 (0/33)
	Miyazaki	2021–2022	0 (0/54)

## Data Availability

The HEV sequences detected in this study were deposited in GenBank (accession numbers: LC857165–LC857174).

## Supplementary Materials

The following supporting information can be downloaded at: https://www.mdpi.com/article/10.3390/v17040524/s1, Figure S1: Geographical distribution of wild boar HEV strains in Japan and in vitro growth kinetics of the wb/Oita/8 strain; Table S1: Detection of anti-HEV antibodies and RNA from wild boars.
