# Supplementary material for: Recent Hepatitis E Virus Infection in Wild Boars and Other Ungulates in Japan
Source: Viruses. 2025 Apr 4;17(4):524. doi: 10.3390/v17040524 (PMC12031028; doi:10.3390/v17040524)
Supplement: Supplementary file 1 [file viruses-17-00524-s001.zip › viruses-3527440-supplementary.pdf]

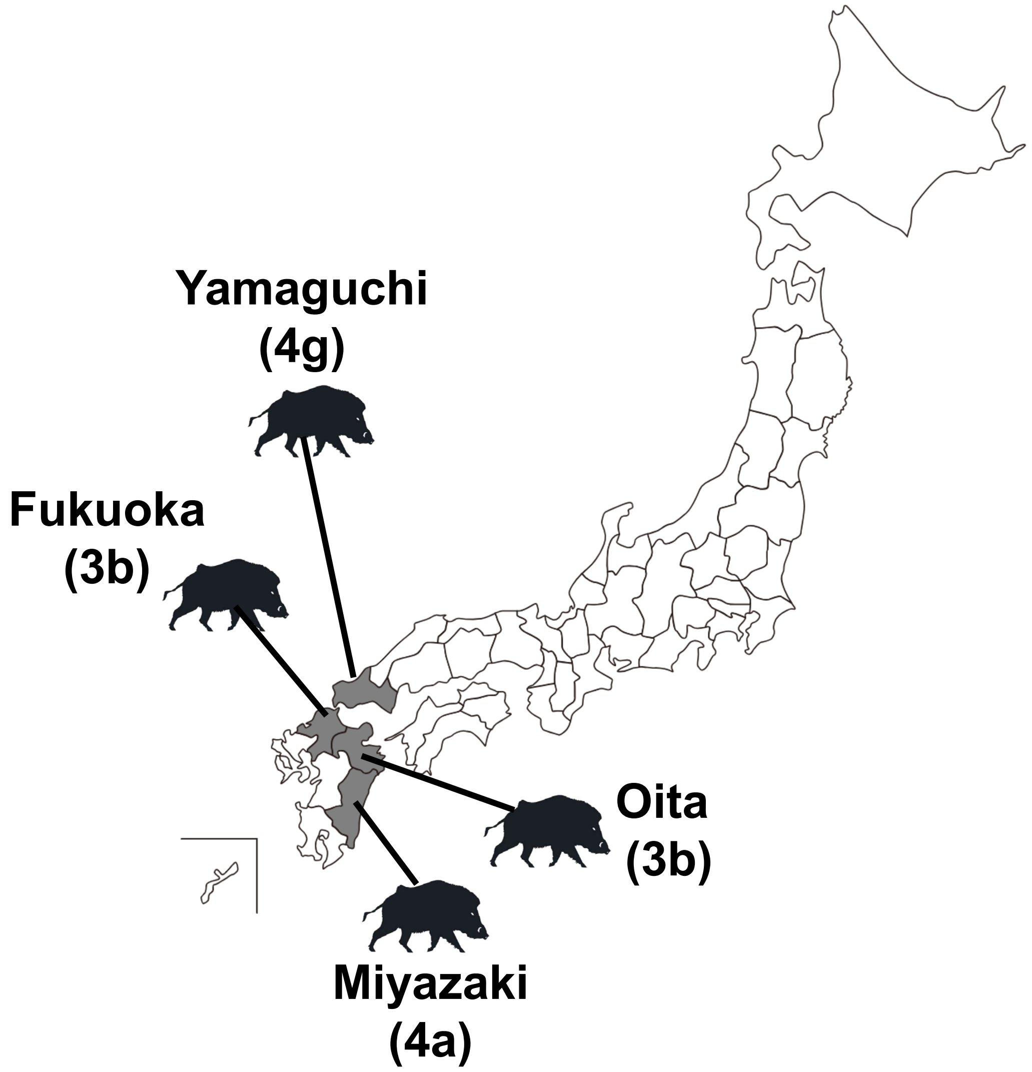

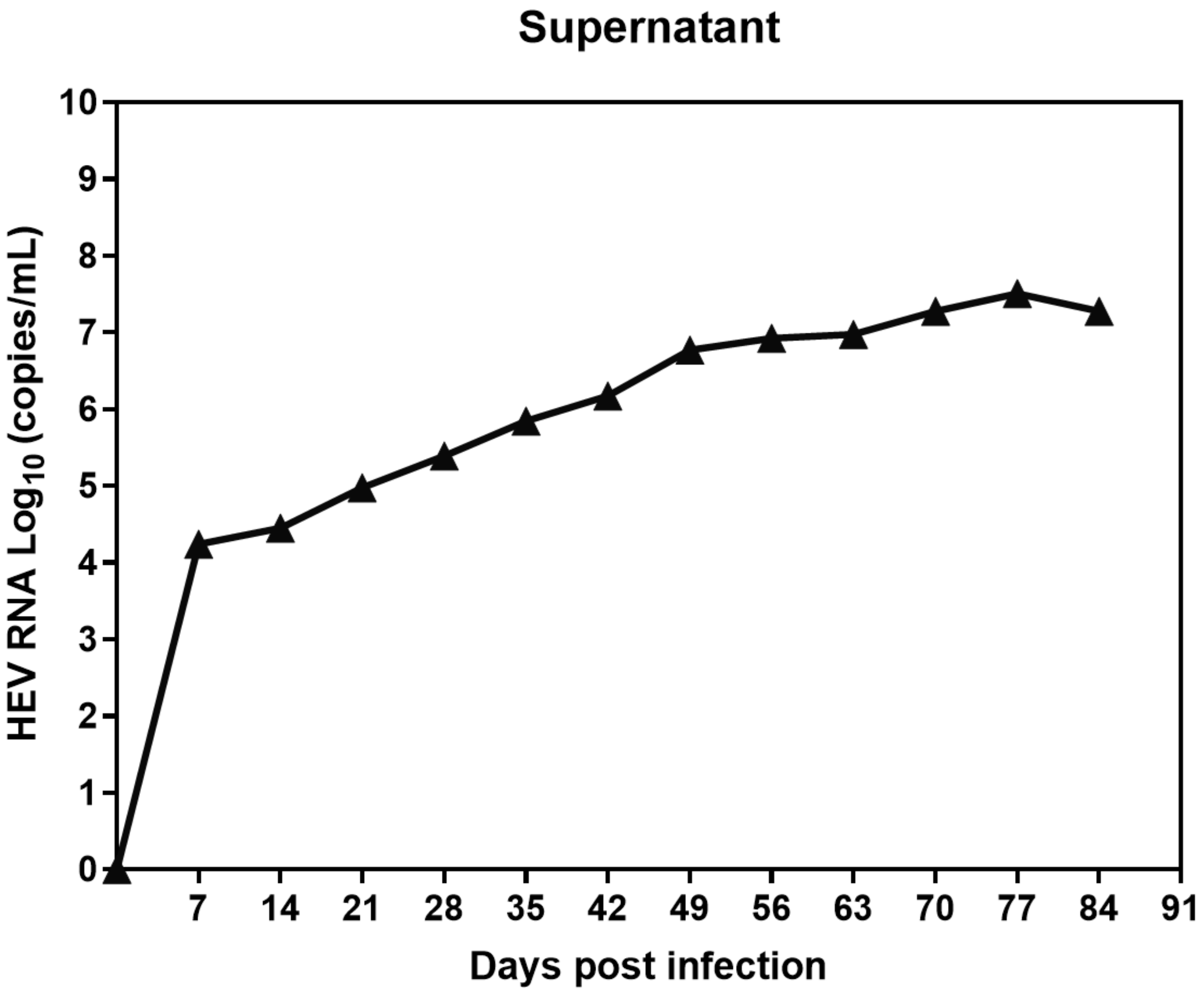

Supplementary Fig. 1. Geographical distribution of wild boar HEV strains in Japan and in vitro growth kinetics of the wb/Oita/8 strain

Supplementary Table 1. Detection of anti-HEV antibodies and RNA from wild boars

		Serum samples						Fecal samples		
		ELISA			Nested RT-PCR			Nested RT-PCR		
		No. of examined animals	No. of positive animals	% of anti-HEV antibody-positive animals	No. of examined animals	No. of positive animals	% of HEV RNA-positive animals	No. of examined animals	No. of positive animals	% of HEV RNA-positive animals
Sex	Male	510	26	5.1	377	0	0	88	4	4.5
	Female	430	23	5.3	315	2	0.6	92	3	3.3
	No record	12	4	33.3	11	1	9.1	6	0	0
Body weight (kg)	<30	98	2	2.0	62	1	1.6	47	2	4.3
	30-50	438	19	4.3	415	0	0	41	1	2.4
	>50	194	15	7.7	186	0	0	21	0	0
	No record	222	17	7.7	40	2	5.0	77	4	5.2
